# Connected Food: First Steps for an Ambitious National Food Strategy

**DOI:** 10.3390/nu16193371

**Published:** 2024-10-03

**Authors:** Neil Bernard Boyle, Victoria Jenneson, Nwamaka Okeke-Ogbuafor, Michelle A. Morris, Selina M. Stead, Louise Dye, Jason C. G. Halford, Steven A. Banwart

**Affiliations:** 1 School of Psychology, University of Leeds, Leeds LS2 9JT, UK; 2 School of Psychology, University of Sheffield, Sheffield S1 4DP, UK; 3 School of Food Science and Nutrition, University of Leeds, Leeds LS2 9JT, UK; 4 Leeds Institute for Data Analytics, University of Leeds, Leeds LS2 9JT, UK; 5 School of Social and Environmental Sustainability, University of Glasgow, Glasgow G12 8QQ, UK; 6 Faculty of Environment, University of Leeds, Leeds LS2 9JT, UK; 7 Australian Institute of Marine Science, PMB 3, Townsville MC, Townsville, QLD 4810, Australia; 8 AIMS@JCU, Division of Research and Innovation, James Cook University, Townsville, QLD 4811, Australia; 9 Food and Environment Institute, University of Leeds, Leeds LS2 9JT, UK

**Keywords:** food system, national food strategy, food policy, food system stakeholders, food system change

## Abstract

**Background:** The global food system faces growing pressure from population growth, climate change, wealth inequity, geo-political instability, and damage to the ecosystems on which our food supply depends. Fragmentation of the priorities and needs of food system stakeholders—citizens, food producers, food industries, governments—compounds the problem, with competing or misaligned interests increasing the risk of failure to adequately meet the needs of those that form, and are served, by the food system. Growing consensus on the need for transformative system level change to address the problems facing the food system is yet to be significantly reflected in strategic action. **Methods:** The national food strategy of the UK is offered as an exemplar to discuss the need to promote more coherent and ambitious visions of transformative change that acknowledge the complexity of the food system as a whole. We draw upon cross-sectoral experience to distil the needs, priorities, and key food system tensions that must be acknowledged to promote transformative systems change that equitably delivers healthy sustainable diets, contributes to a resilient global food system, and protects the environment. **Results:** Greater coherence, ambition, and consideration of the food system as a whole are needed if a UK national food strategy is to contribute to significant transformative change. **Conclusions:** To promote this, we advocate for (1) a food system digital twin to model and test potential food system interventions or legislation; (2) a citizens’ forum to inform and co-develop a cohesive national food strategy; and (3) increased cohesion and integration of food system governance within government to drive a coherent, ambitious national food strategy.

## 1. Introduction

Insecure access to food is growing across the globe due to fragmentation in the global food system driven by social division and food system stakeholders with deeply vested interests in producing, supplying, and accessing human nutrition. Failure to develop and implement cohesive food strategies that can deliver access to affordable nutritious food will widen the already growing gap in social and economic equity. At a global level, there is broad consensus on the U.N. Sustainable Development Goals (SDGs) to achieve a peaceful and prospering planet [1]. Food is at the centre of these goals, spanning food security, dietary health, environmental sustainability, and economic development. However, there is a significant gap between these international strategic goals and the governance and practice required to achieve them at the community, regional, national, and international levels.

The recently ousted UK Conservative Government’s national food strategy, which outlined plans for the food system in England, is a case in point. The Government Food Strategy Policy Paper [2] (hereafter, the GFS) has been criticised for lack of strategic cohesion, as well as failing to acknowledge the complexity of the food system as a whole [3]. We propose it also failed to adequately address the distinct needs and priorities of the stakeholders of which the food system is formed, and whom it serves. We address this UK ‘strategy gap’ by acknowledging the competing interests and outcomes within food supply systems and identifying value capture beyond monetary return on investment. As we move into a new political era under the newly incumbent Labour Government, we recommend steps to engage currently competing interests in tackling systemic barriers to positive change, including data-driven transparency in the food system, coherent policy across sectors, and a citizens’ forum to give voice to national ambitions for what the UK food system can deliver. 

Transforming the global food system and its impact at a household level is an essential step in achieving strategic national priorities for the UK and globally. The barriers are substantial. A growing proportion of the global population suffers from obesity and diet-related disease [4]. Wealth inequality traps the most vulnerable into consuming poor diets, having to prioritise cheaper food for hunger alleviation over the relatively higher cost to access food that provides adequate nutrition [5]. This insecure access to adequate diets can lead to the triple burden of malnutrition, with increased risk of undernutrition and, paradoxically, overnutrition, which is associated with a greater risk of weight gain and obesity alongside the micronutrient deficiencies that characterise undernutrition [6,7]. 

Food supply contributes one-third of human greenhouse gas emissions, 71% from agricultural production [8]. Despite one-third of food produced being lost as waste [9], the continued transition of forest and grassland to intensive agricultural production is the leading driver of global habitat destruction and accelerating decline in biodiversity [10]. The Sustainable Food Trust’s ‘The Hidden Cost of UK Food’ report [11] estimates that every GBP 1 spent by UK food consumers is associated with an extra GBP 1 in hidden food system-attributable cost due to environmental impacts, dietary-related ill health and lost labour capacity, and agri-food subsidisation costs. In simple terms, the market price of food is too cheap to cover the hidden costs, and nutrition is too expensive to be universally available.

Solutions are not simple. The pressures on supply chain businesses are enormous, with financial margins competing to generate return on investment. This commercial pressure is occurring at a time of increased geopolitical uncertainty and commodity price fluctuation, when household incomes are shrinking due to unexpectedly high global inflation. The projected impacts of climate change contribute to market uncertainty. These events and more frequent and greater weather extremes affect the predictability of food production, logistics, price, and availability [12,13]. Protecting the immediate monetary value of supply chain businesses puts downward pressure on the contract price for agricultural production and upward pressure on the market price of food products. These pressures adversely impact farmer livelihoods and household finances upon which agri-food businesses depend.

The profound system shocks of the COVID-19 pandemic and Ukraine conflict have further exposed the vulnerability of the global food system [14]. The impacts have underlined the need and urgency for transformative action to build a resilient and sustainable food system; one that delivers access to healthy, secure food and fair livelihoods for those creating value. This includes value traded in the market and value that is hidden but contributes to the full value of nutrition and nature. Addressing this full range of interests, both when conflicting and aligned, is essential in national food strategies and their policy implementation and governance. 

A major independent review of the UK food system was commissioned by the Conservative Government in 2018 to aid the creation of the country’s first national food strategy for 75 years. Its purpose was “to address the environmental and health problems caused by our food system, to ensure the security of our food supply, and to maximise the benefits of the coming revolution in agricultural technology” [5]. The two-part National Food Strategy independent review (hereafter, the NFSIR) outlined the role the UK food system plays in detrimental impacts on human health and the environment and offered 14 coherent and well-reasoned recommendations to address these food system impacts and move towards a better food system. 

The Conservative UK Government’s response to the NFSIR outlined in the GFS largely ignored many of the recommendations and was particularly deficient in acknowledging the need for systems thinking to affect transformative change [3]. To address these weaknesses, we argue that a national food system must be treated as strategic national asset, acknowledging its role not only as a crucial economic asset, but also as a key source of national nourishment, security, identity, and social and cultural value. Such a crucial asset requires substantial and continuous cross-sector effort to assess, monitor, protect, and improve. A national food strategy offers a significant opportunity to transform the food system and maximise the potential to safeguard and promote healthy and sustainable food for all its citizens, to support employment and livelihoods, and contribute to a resilient global food system that protects the ecosystems on which it depends. 

As a starting point, the interests of key stakeholder groups in the UK food system must be clearly acknowledged, spanning those from farming and fishing through to household food use and resource recovery. These interests, in the face of systemic change, may be in conflict, non-aligned, or in synergy. Agreeing the need for transparency in interests and related value, both market and hidden, is an essential first step.

To illustrate this approach, we use our collective cross-sectoral experience to describe the interests and responsibilities of four key stakeholder groups within the food system: citizens, farmers/producers, the food industry (both manufacture and retail), and policy-makers (national and local across all departments with food-related responsibility). We distil this down to present a non-negotiable need for each stakeholder and argue that if any one of these groups’ needs is not met, then the food system has failed. Throughout, we also advocate for the natural environment on which we are profoundly dependent upon to meet these needs. We propose key system tensions between the needs of stakeholders and the imperative to build environmental value that will last into the future. 

We argue that the GFS failed to meet the needs of key stakeholders and we call for greater ambition, focus, and coherence to ensure these needs can be addressed while building value beyond that captured in conventional financial accounting (e.g., natural capital) and which is strategic to long-term national interests. Practically, we draw upon our domain expertise to advocate for three pathways of action, which would go some way to addressing the current system failures. These are (1) a food system digital twin which brings together a variety of data sources from across the food system in which to visualise patterns from and to test potential food system interventions or legislation upon; (2) a citizens’ forum with a focus on co-developing a cohesive national food strategy; and (3) better integration of national government departments to improve the scope and scale of a more ambitious national food strategy.

## 2. The Food System

A food system comprises the total value-creation (both monetary and non-market) of interconnected activities of all stakeholders that contribute to the production, aggregation, processing, distribution, manufacture, retail, consumption, disposal, and resource recovery of food [15]. Food systems exist, operate, and interact at the global, regional, national, and local levels, with location-specific characteristics and practices defined by geography, tradition, culture, wealth, and the local natural environment. Such complex sets of activities and interactions can only be understood by approaches that acknowledge the interconnected, dynamic nature of the system as a whole. A systems approach includes the macroeconomic inputs and uses of labour, finance, expertise, and material resources that contribute to value creation of food products, dietary health, environmental stewardship, and the recovery of material resources after food use. 

The purpose of this broad view of the food system and its interacting parts is to understand the sources and propagation of risks (e.g., hunger, malnutrition, social instability, economic damage) and to identify points of effective intervention where purposeful action can propagate and amplify positive impact. The aim is to avoid food system failure and the devastating consequences that can follow, to build resilience and sufficiency of nutrition for all people, to mitigate future adverse impacts, and to create strategic value that endures for future generations.

Systems approaches promote consideration of the breadth of outcomes of food systems, such as health and nutrition, livelihoods, and environmental impacts. As with any complex system, there will be alignments and misalignments between the diverse, and at times, competing, needs and interests of a constellation of stakeholders. Not all actions taken to transform the food system will produce a ‘win–win’ outcome; trade-offs and compromises will have to be identified and addressed explicitly. For example, action taken to protect ecosystems may have initially unpopular repercussions with respect to food choice and availability. In addition, action required to ensure affordable access to nutrition may require commercially challenging restructuring of food product pricing. We must acknowledge these complexities if we are to have an impact on achieving cohesive and realistic transformative change in the food system. 

Given the profound potential consequences of loss of food security, and the clear strategic national priority to maintain resilience of access to nutrition, it is imperative that the responsibilities of the different stakeholders are integrated and potential points of failure identified. This is so resultant actions can be tailored to the local specific context to facilitate uptake of any measures introduced. We argue that the stakes are so high that, if needed, stakeholder responsibilities to avoid failure must be prescribed/compelled and/or incentivised with clear and consistent goals and the required action and legislation.

## 3. Examining Food System Stakeholders

Each of us is a stakeholder in the food system and may be represented by one or more stakeholder groups. We can each benefit from a well-functioning food system or suffer because of system failures. We argue that a successful food system should maximise the benefits to all system stakeholders and not discount or disadvantage any group of stakeholders in favour of another if equitable access to food security is a key goal. 

### 3.1. Citizens

The majority of the UK population has easy and consistent access to a wide variety of foods, independent of seasonality and the capacity for foods to be cultivated in the UK. This requires a global food system that consistently produces and transports food at significant environmental, economic, and social cost. Citizens also expect food, particularly certain staple foods, to be affordable. Expectations of food affordability are often at odds with sustainability; producing foods more sustainably can increase the cost of production and cost for the consumer. Food retailers also operate on the assumption that the majority of citizens expect food to look a certain way, which can have significant waste implications.

For decades, the food system has been very effective at quietly meeting these demands. The fragility of the food system was laid bare by the COVID-19 pandemic and the Ukraine conflict as food supply chains laboured under the strain of these external shocks. The perceived shortage and significant increases in food cost has thrust the hitherto hidden operations of our food system to the fore as these global shocks test the resilience of food chains. Reports of sale rationing and shortages of certain foods in the UK in 2022 hint at a system that is experiencing difficulties maintaining the easy and consistent access to the array of foods to which we are accustomed. Despite such recent shocks, the UK food system has demonstrated resilience, and disruptions were small and short-lived. Yet we are perhaps guilty of complacency and assume the food system will always be able to iron out these system malfunctions; perhaps a precarious assumption considering the UK’s reliance on food supply from climate vulnerable regions [16]. We, as food consumers, must accept some responsibility to adapt our expectations and behaviour to make a significant contribution to a healthier, more sustainable and resilient food system. However, established behaviour patterns are difficult to change; especially ones that are easy, convenient, and taste good.

The historical government policy focus on placing responsibility to consume a nutritious diet at the level of the individual has not worked and cannot be expected to work in food environments that contain barriers to healthy dietary choices and, in some cases, promote poor dietary choices. At the heart of the NFSIR was the proposition that the food environment is making us ill. The pervasiveness of cheap, energy-dense, highly palatable food hinders consumer capacity to make healthy choices and consume the nutrients needed to support healthy life. Moreover, consumers are making choices in a system in which highly processed, unhealthy foods are often cheaper than healthier foods [5]. The proportion of adults in England who were living with overweight and obesity rose from 52.9% to 64.3% between 1993 and 2019. In the same period, the proportion of those who were living with obesity rose from 14.9% to 28.0%. One in ten children is living with obesity by age 5, rising to 23% by 11 years old [17]. A large proportion of citizens are failing to consume diets recommended for health and longevity despite significant investment in public health campaigns—adherence to the Government’s Eatwell Guide diet is startlingly low [18]. It could be argued that other stakeholders in the food system should take steps to mitigate against the apparent reluctance of a large proportion of citizens to eat a healthy, balanced diet (e.g., the soft drinks industry levy, which has been associated with a decreased prevalence of obesity in children [19]).

Individual responsibility implies food consumers have a degree of choice in the diets they consume. For a growing proportion of the UK population facing food poverty, dietary and sustainability choices are significantly restricted by both impoverished food environments and food affordability. Meeting the Government recommended Eatwell Guide would cost the poorest fifth of UK households 50% of their household disposable income, compared to 11% in the richest fifth of households [20]. Children in the lowest income decile are nearly twice as likely to be living with overweight or obesity by age 11. Those in the two lowest deciles consume ~29% less fruits and vegetables per day compared to those in the highest two deciles [5]. This disparity in diet quality is expressed in impoverished life outcomes, including lower life expectancy and earlier onset of ill health [21]. A food system must deliver equitable, affordable access to nutritious food. In its current form, the UK food system is falling short for too many citizens. A national food strategy is a timely opportunity to tackle the underlying causes of household hunger and malnourishment in our communities. 

Citizens do not merely operate as consumers in the food system; in addition to sustenance, the food system is a source of livelihood for many. In early 2022, the agri-food sector in Great Britain employed 4.1 m people—13.4% of GB employment [22]. In 2021, 22% of workers in the food system earned the National Minimum Wage or below, compared to 8% of workers in the UK economy as a whole [20]. A thriving food system needs investment in food education and well-paid jobs to make the most of this important sector of the UK economy. 

For the UK’s Black, Asian, and minority ethnic (BAME) citizens—who make up close to 15% of the UK population—food is not only a source of nutrients; it is intrinsically linked to identity and culture. However, the dietary needs of BAME groups are often overlooked; UK supermarkets do not widely sell BAME cultural foods [23,24], and both the NFSIR and GFS fail to acknowledge the UK’s ethnic food diversity. Access to affordable, quality food has remained a continuing challenge to BAME groups, who are also disproportionately disadvantaged by health and income inequality [25]. Presently, BAME groups depend on independent shops catering to specific cultural preference, where foods are expensive and sometimes of low quality and labels do not reveal sufficient information about production ethics. An ethnically diverse country like the UK needs to demonstrate in its national food strategy how minority ethnic groups can access quality cultural food at an affordable price with no or minimal environmental cost at home or abroad, depending on from where the foods are sourced.

**Citizen non-negotiable:** Access to affordable food that meets dietary preferences and jobs that permit a decent quality of living.

**Key system tensions:** The demand for consistent access to a wide variety of food at low cost is at odds with sustainable use of our ecosystems. A considerable proportion of the population are also consuming low-quality diets with significant dietary-related health impacts; the food system and food environments often promote this consumption pattern. A rising proportion of the population are also unable to reliably afford a high-quality diet.

### 3.2. Farmers/Producers 

The UK sources about half of its food from the national farming sector [26]. Agricultural production in the UK is highly fragmented; there is no single dominant business model. Farmers in lowland areas may prioritise arable agriculture and intensive crop production to maximise yield and gross income, while hill farming may focus more on livestock production and lower operating and capital investment costs to maintain livelihoods. Some UK farmers focus on relatively smaller volume and higher market price through defensible claims of exceptional animal welfare and environmental stewardship. Others maintain high volume using conventional farming practices that are well understood and predictable in terms of costs and production level. 

Farming in the UK in the past two decades has stagnated as a business proposition. During this period, average income for individual UK farms has remained flat at around GBP 50,000 per year [27], while commodity prices prior to the Ukraine conflict were largely stagnant [27]. In the farming sector, over a third of all farm holders in England were over the age of 65 years in 2016; just 2% of holders were less than 35 years old [27]. This is not an economically sustainable production base. If food and nutrition are so precious, why are farmers having to operate within such thin and uncertain margins, often whilst having to service substantial capital debt? Taken at face value, the UK does not value farming as a primary economic sector, neither in policy terms nor in the prices paid to producers.

Departure from the European Union and the Common Agricultural Policy (CAP) exacerbates the perception of a policy vacuum, with the certainty that the post-Brexit national operating environment is changing rapidly but without clear priorities or articulation of a new, coherent policy destination. This is compounded by competition from imports mediated by new free trade agreements with uncertain consequences for UK production, food quality and food availability [28]. There has been clearly articulated government ambitions on public payments for public goods, particularly environmental stewardship through Environmental Land Management (ELM; [29]) with payments through a range of mechanisms of the Sustainable Farming Incentives (SFIs; [30]). These schemes address mitigating climate change by carbon sequestration in vegetation biomass and soil, ecosystems restoration, and animal welfare. The potential public benefits are well understood [5]. The ambition is laudable and clearly in the public interest. However, for farmers attempting to navigate the changing geopolitical environment, UK payment schemes have not always been rapidly nor clearly implemented. In many cases, payments are likely to be well below that which many individual farm businesses were receiving through the CAP. Indeed, initial enrolment on the schemes has been low [31]. On the other hand, UK food industry purchasing responses to trade agreements are immediate and may feel threatening to UK producers. 

The business environment for farmers is currently communicated through competing voices, including (1) current market volatility of input costs for fuel and fertiliser, (2) rising costs of commercial borrowing, (3) uncertain commodity prices during a European military conflict, (4) new UK free trade agreements, (5) rapid changes in import and export markets, (5) implementing SFIs, and (6) the national food strategy for England. How can the farming sector navigate such a complicated environment?

We argue that farmers in England and across the UK want fair prices for what they produce and a business environment that is stable for (1) the costs of their inputs and (2) the price of the contracts with buyers. They want more power in setting a fair price of their contracts. It is simple if farmers are only expected to maximise yields and sell at market prices, but farmers are being asked to do so much for the nation—produce food that looks appealing, tastes delicious and is nutritious, all while delivering high standards of animal welfare, keeping the countryside attractive to weekend visitors, reducing or eliminating pollution, and they are now under pressure to save the planet from climate change. Farmers are expected to deliver these public goods on a shoestring budget relative to the effort required, and without increases in the cost of food. If manufacturers, retailers, consumers—and politicians—want a better world for food, then they must negotiate the price of this action with farmers.

**Farmer/producer non-negotiable:** Power to negotiate market prices that reflect the importance of public good and the demand from retailers and food manufacturers for supply chain transparency, justified claims of environmental benefits of good farming practices, and ethical sourcing.

**Key system tensions:** Relatively small geographical production base set against the national demands of a G7 UK economy, with the resulting trade pressures from imports at odds with the UK ambition for geopolitical independence and control over national food security.

### 3.3. Food Industry

Food businesses are a major part of the UK economy, with more than 13% of people employed in the agri-food sector that contributed GBP 116.2 bn. to the national gross value added in 2020 [22]. Thus, food industry profitability is important for maintaining a healthy job market and household incomes. However, our vibrant and competitive food sector brings about problems in and of itself. While the vast amount of choice on the market allows customers to vote with their food choices, it leaves food businesses vulnerable and can promote undesirable practices. With food prices growing at their fastest rate in 40 years, food businesses attempt to shelter customers from disrupted supply chains and increased costs to remain competitive, but the capacity of businesses to absorb increased costs is limited and affects profit and loss balance as well as shareholder dividends. The equitability of cost absorption across food businesses has been the source of recent tensions between retailers and manufacturers, with large supermarkets holding the balance of power; six supermarkets held 81.1% of the UK grocery sector market share as of October 2023 (in order of share: Tesco, Sainsbury’s, Asda, Aldi, Morrisons, Lidl; [22]), and adding The Co-op and Waitrose takes the share to 92.3%. The out-of-home sector is particularly vulnerable, and rising costs have led to large numbers of pubs, restaurants, and cafes closing.

To compete, food businesses have to adapt to be more agile to customer demands, with rapid product development and industrial operations allowing them to benefit from efficiencies and economies of scale. However, these benefits can be counter to equitable contract terms with producers and suppliers. Furthermore, recent system shocks have exposed the fragility of our food industry’s just-in-time supply chains that require complex logistics with less capital investment in storage capacity and lead to commodity shortages and increased costs for raw ingredients and energy.

Manufacturers, distributors, and retailers support a free market for foods in which consumers are free to choose what they wish in accordance with their values and what they can afford. The ability to provide customers with what they want can give food businesses a competitive edge and has led to huge investments in market research and the business of selling market insights to remain up to date with customer trends. Meeting the desires of consumers has also resulted in a huge expansion of food products on the market over the last few decades, with the large supermarket retailers now offering tens of thousands of product lines at any one time. While this has some benefits for the availability, affordability, and diversity of the products on offer, this rapid range expansion has also led to a diversion of the food market offering away from dietary recommendations towards nutritionally dense/nutrient poor foods that are convenient, highly palatable, and cheaper to produce. In population health terms, we consider this a failure of the free market. Highly palatable foods high in fat, sugar, and salt (HFSS) are estimated to make up around 40% of the UK retail product market [32], despite recommendations to limit their intake. Aside from being merely responsive to consumer demand, the food environment itself serves to shape customer wants and expectations, creating a reinforcing loop.

Meeting customer needs for affordability and alignment with values have become selling points upon which businesses may compete and profit. Yet free-market principles bring about tensions between the cost of goods and the ability to deliver ethically aligned values. Products that are fair trade, organic, and environmentally sustainable carry a higher price point due to increased downstream production costs through, for example, higher worker wages; lower yields because of less-intensive farming practices; and reduced sourcing agility. At the same time, industrial efficiencies and economies of scale have driven down prices for processed foods. While this has benefits for food availability, it has implications for the ethical and nutritional characteristics of products. These tensions have created a market in which products that serve the low-cost need sit at the opposite end of the spectrum from those that adhere to ethical values. Thus, the food industry has shown that it can serve the needs for low cost and ethical values independently, but there is increasing pressure upon it to address both paradigms simultaneously.

While the food industry tends to oppose legislation that interferes with free market principles, there is a growing appreciation for the role of Environment, Social, Governance (ESG) standards to align brand visions with positive social messages to add value and resonate with customers. This comes with an increasing understanding within businesses of societal challenges and desire to act in the public interest. It is important to note that the food industry is not opposed to increasing the health and sustainability value of products. Indeed, there are notable examples of positive voluntary industry commitments, including the Public Health Responsibility Deal (https://www.gov.uk/government/news/public-health-responsibility-deal, accessed on 8 January 2024), the Action on Fibre initiative (https://www.fdf.org.uk/fdf/what-we-do/diet-and-health/action-on-fibre/, accessed on 10 February 2024), and pledges to adhere with the proposed ban on price promotions for HFSS foods, despite delays in legislation implementation. Yet voluntary action has been limited in its effectiveness within such a competitive market. Those companies that follow ethical principles may ultimately lose out on profits and market share, disincentivising ESG practices. As a result, there are now calls on all sides, including industry actors and representative trade bodies, for clear legislation to ensure equity across competition. The desired level playing field would see responsible industry practices become the norm rather than ‘value added’ which may be undercut and undermined. The GFS’s plans for mandatory data reporting within the Food Data Transparency Partnership will provide a positive step but will not override profits and return on investment as industry’s key performance indicator. Furthermore, it is unclear whether the Food Data Transparency Partnership would extend to nutrition and health metrics. More substantive policy levers that are needed to realign the food system with ethical values have been ignored by the GFS. 

**Food industry non-negotiable:** A profitable food industry that meets customer needs.

**Key system tensions:** The costs of delivering ethically aligned values whilst remaining profitable and meeting customer needs for affordable foods. 

### 3.4. Policy-Makers

Policy-makers are faced with multi-faceted external pressures: from the food industry, which is faced with reduced purchasing power of consumers and high energy and food commodity prices; from voters experiencing rapid food price inflation and lower household living standards; and from the health services tasked with managing the national burden of the long-term impact of poor dietary health. These recent and rapid changes present unprecedented challenges to maintenance of trust from society especially in food security that is affected by the transition in trade policy post-Brexit, the supply gaps experienced throughout the COVID-19 pandemic, and price inflation during the ongoing Ukraine conflict. Keeping voters and investors happy is not easy in the current political climate. These challenges and the dependencies between them emphasise the need for a systems approach to co-developing national food strategies that enables evidenced science policy to link priorities of government, industry, and civic society. 

As highlighted in the NFSIR, the responsibility for food policy in England is spread wide across government departments [33]. National policy bodies such as The Department of Business and Trade need to help keep the food industry sustainable with policies to encourage inward investment, employment, and vibrant trade relations that offer access of UK farming and food exports to international markets and access to good quality, cheap food imports to keep prices low for consumers. The Department of Science, Innovation and Technology is tasked to improve skills to support industry innovation and better jobs for the 13% of the UK workforce in the agrifood sector. The priorities of the Department for Environment, Food and Rural Affairs are to make sure everyone’s food is safe and nutritious, that the farming sector remains economically strong, and that the environment is protected from the impacts of food production and enables the UK production of high-quality food crops and livestock. The Food Standards Agency leads on ensuring the regulation of safety, composition and hygiene of food. The Department for Energy Security and Net Zero wants to make sure we take steps towards a net zero food system. The migration of labour to contribute to the operation of food system is controlled by the Home Office. The Department for Digital, Culture, Media & Sport (replaced by the Departments for Science, Innovation and Technology and for Culture, Media and Sport) had main oversight on food advertising. The Department of Health and Social Care—and the Office for Health Improvement and Disparities—wants to reduce the burden and cost of healthcare through improved dietary nutrition and reduced obesity and malnutrition. The diverse range of departments involved in food make co-developing policy challenging thus leading to unintegrated strategy. 

Multi-level governance—local, regional, national, and international levels—is required to implement national strategy through local government action that addresses communities’ needs and to align national strategy with the discharge of UK obligations to intergovernmental treaties and U.N. Conventions. The Department for Levelling Up, Housing and Communities (renamed Ministry of Housing, Communities and Local Government [MHCLG] by the incoming Labour Government) can implement policy that supports local government and the communities they serve. The priorities include equitable access to affordable nutrition that also results in improved health and social outcomes, with broad economic and social benefits with a healthier, more productive workforce. The Foreign Commonwealth and Development Office can project improved national strategy and implementation through UK national policy interventions. These efforts can positively influence international agendas and support the U.N. 2030 Agenda and the achievement of SDGs. Food systems are particularly relevant to SDG 2 Zero Hunger, SDG 3 Good Health, SDG 5 Gender Equality (through maternal and infant health), SDG 13 Climate Action, SDG 14 Life on Land (through land use) and SDG 15 Life Under Water (protecting ecosystems for fisheries). Agriculture and food systems are particularly important sectors in achieving the global benefits articulated in treaties and U.N. Conventions on Human Rights, Climate, Biodiversity, Land Degradation and the U.N. Protocol on Water and Health.

**Policy-maker non-negotiable:** Clarity of decision making on different policy areas across government departments that are in synergy and in conflict regarding national food strategy.

**Key system tensions:** Tensions between free trade and geopolitical independence, between farming production costs and retail prices, between food prices and the costs of environmental and human health impacts.

## 4. Reflections on the Government Food Strategy

A national food strategy offers a significant opportunity to transform the food system by laying out a clear and cohesive strategy for implementing change. This must be undertaken within the priority frameworks of food security, protecting the environments on which our food system depends, and stimulating significant advances towards resilient, equitable access to healthy, nutritious food for all. If we are to fulfil our obligations to the Paris Agreement and SDGs, we need to meet these huge challenges with considerable coherent and concerted action—in its present form, the outgoing Conservative GFS falls appreciably short in terms of ambition, urgency, and coherence. Indeed, it is arguable whether the GFS is a strategy at all—a concern voiced by the author of the NFSIR [34]. It is difficult to discern new policy measures from existing ones in the GFS, which reads more like a list of aspirations rather than clear, coherent policy measures. The Conservative Government acknowledged as much: (the GFS) “is not a comprehensive summary of everything that government is doing to improve our food system, or the actions being taken by industry and other key actors. It instead articulates some of the key priorities for action within our food system…” (GFS; Introduction; point 8; [2]). Indeed, the previous Government’s position that the existing powers in primary legislation would be sufficient to implement the strategy did not hint at transformative action [2].

We acknowledge that the formulation of a national food strategy is an ambitious undertaking that needs to balance the complex, often competing, interests of stakeholders in the food system. However, the strategy proposed by the outgoing Government is lacking in new clear, coherent, and integrated actions and policies; rather, it lists a collection of siloed expressions of intent to consult or deference to existing or prospective policies (e.g., Environment Act, Fisheries Act, Levelling Up White Paper, Land Use Framework). The lack of systems thinking in the GFS perhaps felt all the starker coming as it did in the wake of the NFSIR, which was punctuated with more nuanced consideration of the food system as whole. The GFS largely ignored the NFSIR and failed to directly address the fourteen recommendations or seventy specific actions it laid out—only one recommendation was fully addressed in the GFS. The failure to respond directly to the recommendations was particularly frustrating since it is reported that the analysis exists within Whitehall [35]. The UK needs a food strategy that provides a coherent framework to form a steady direction of travel rather than oscillating between loosely related policies. 

All stakeholders in the food system have a responsibility and a role to play in making significant progress towards food system transformation. However, given the complexity and fragmentation of interests in the food system, a strategic overall responsibility should lie with government to enact ambitious and coherent, systems-based policies to address and balance the needs, interests, and actions of stakeholders across the food system to support transformative change. It is reasonable to counter this proposition and, for example, argue that responsibility for tackling obesity lies with the food industry and the food choices citizens make. However, there is little to suggest citizens will abandon unhealthy, unsustainable dietary habits, and the food industry faces losing competitive edge by voluntarily acting to increase the health and sustainability of products without clear legislation to ensure equity across competition. The GFS does emphasise a shared responsibility to identify solutions to food system problems—such as obesity—acknowledging the need to “create the right incentives for change. This includes a role for government in creating a level playing field through regulation” (Chapter 2, point 2.1.8; [2]). However, in its’ current form, there is limited stated commitment to significant regulatory action in the GFS compared to the recommendations put forward by the NFSIR.

High-level food system interventions that restrict or disincentivise activity are at odds with the consumers’ desire for choice and a government’s likely reluctance to be seen to overtly constrain the choices of its electorate or interfere with the free market activity of the food industry. This may account for the absence of any significant policy to implement food environment change in the GFS (e.g., the rejection of the proposed tax on sugar and salt). Yet there is evidence to suggest the electorate support concerted action on key issues such as obesity [36]. Some problems may be just too imperative to leave to the self-regulation of markets. The impacts of the smoking ban highlight the success possible if fear of accusations of ‘nanny stateism’ are overcome. 

Whilst the previous Conservative Government had slightly shifted its narrative by acknowledging the role the ‘junk food cycle’ may play in promoting poor dietary health (the Conservative Government’s July 2020 Obesity Strategy acknowledged the role of environmental factors; [37]), a focus on individual responsibility prevails still. This is despite a 30-year history, and 14 governmental health strategies stating 698 recommendations, emphasising self-responsibility to target obesity reduction failing to halt the rising obesity crisis [38]. Crucially, the GFS did not explicitly address the issue of health and food or identify any actions to address the junk food cycle.

Action on the junk food cycle and food environments had been deferred to the DHSC White Paper on health inequalities; this White Paper has been repeatedly delayed and subsequently shelved in January 2023. Legislation preventing the promotion of unhealthy diets to children and the ban on volume price promotions of HFSS have also been repeatedly pushed back without clear justification. In the meantime, the health of the UK is in decline [39], inequitable access to quality nutrition is increasing, and diet-related health inequalities are widening [20]. Bold action is needed to ensure the prioritisation of health and sustainability—and equitable access to both—are embedded as norms in the food system and expressed in food environments, rather than selective value adds by industry who may feel curtailed by an unequal legislative playing field. The paucity of ambitious, transformative strategies in the previous Government’s approach makes empty rhetoric of claims of being able to meet previous commitments to halve childhood obesity by 2030, reduce the healthy life expectancy inequity gap by 2030, and add five years to healthy life expectancy by 2035. 

Complex challenges such as the junk food cycle require clear statement of current knowledge on the systemic roots of the problem. This understanding can be checked against evidence, and interventions constructed at pilot scale that can be tested, adjusted, and confirmed for effectiveness before scaling. As an example, we consider the previous proposition that food is too cheap and nutrition is too expensive. We can test this statement by comparing the price, sales volumes, and nutrient content of food purchased for home consumption and the economic and social position of groups making the purchasing choices. We argue that consumers with less economic power select the lowest-price foods with the highest energy content, which prioritises immediate hunger alleviation at the lowest cost to a household, and that the selected foods largely fail to meet nutrition requirements for good dietary health. Alternatively, we can identify if purchases that provide hunger alleviation with high nutrient content are indeed more expensive and quantify the price gap to be covered that will allow for hunger alleviation while meeting nutritional needs. 

Outcomes from the evidence might indicate that price structures need to change in order to deliver improved dietary health at population level. It might be that junk foods with high energy and low nutrient content require a higher price point that results in lower volume of sales. In this case, the increased price on the volume of junk food sales might be used to offset the cost to households of nutrient-rich foods such as fresh fruits and vegetables and high-fibre bakery products. The cost of non-essential gentrified food that is preferred by higher-income groups may then need to increase further to provide sufficient operating margins for supply chain businesses. The aim would be to place better nutrition within the financial reach of households that are otherwise priced out of nutrition and to reduce the consumption of junk foods that more likely contribute to the double burden of obesity and poor nutrition. 

We are not advocating this specific solution, but we aim to illustrate the need for evidence-based systems thinking to identify connections between drivers of behaviour and the outcomes for affected groups and pathways and barriers to positive change. This approach then identifies points of intervention and risks from implementation to be transparently acknowledged and mitigated. We argue that food system complexity, due to the fragmented sectors that supply and use food and the competing interests of the groups affected, are preventing action to tackle challenges such as the junk food cycle and preventing a systematic analysis of what steps are needed for positive change.

A great deal of the burden of transformative change in the food system has been laid at the gate of the farming industry, which is already under significant strain. Protection of the environment seems to rely upon incentivising farmers and producers to adopt more sustainable practices—whilst maintaining the quantity, quality, and affordability, and simultaneously increasing the healthiness, of their produce. The feasibility of this, and a coherent strategy of how it will be achieved, is at present, somewhat lacking. Existing, distinct policy frameworks have been referenced as sufficient to meet these significant challenges, supported by changes to funding for farmers that promote sustainable production. Productivity gain has also been referenced without any further clarification. Maintaining production levels and simultaneously increasing sustainability will clearly require new, innovative approaches to producing our food. 

Whilst falling somewhat short of the GBP 1 bn. investment in innovation proposed in the NFSIR, the previous Government did acknowledge investing in innovation as a key pathway to meet these challenges. For example, innovations evolving from a large Farming Innovation Programme were planned to be disseminated to farmers by a What Works Centre to promote on-farm adoption [2]. A suite of food system interventions has been funded to deliver and evaluate interventions to promote healthier and more sustainable diets (e.g., https://www.salientfoodtrials.uk/, accessed on 23 June 2024). The much-anticipated Sustainable Farming Incentive scheme has been revealed (although not fully) after some delay and confusion [30]. The principle of using public money to support public goods has to be considered a forward-thinking step and has been welcomed as broadly positive. The success and usability of the system, and accessibility to the scheme across the farming industry, will become clearer over the coming months as the system is introduced in a staggered fashion. It is crucial such initiatives not only deliver on environmental goals but also on farmer’s futures and farming as a business proposition. 

The insight and innovation from these activities can play a key role in meeting the food system challenges. However, it is not clear how these activities and innovations are integrated to give insight at a food system level. The complex activities and interactions in a food system can only be understood by considering the interconnected, dynamic nature of the system as a whole. What potential alignments and misalignments between the diverse, and at times, competing, constellation of food system stakeholders will result from these research innovation outputs. A system oversight is essential to model the impacts of food system interventions to identify the sources and propagation of risks, and to identify points of efficacious intervention. We propose three actions that can promote a system approach giving greater oversight of the food system and provide valuable stakeholder insight to support delivery of a transformative national food strategy.

## 5. Next Steps for a Transformative Food Strategy

### 5.1. Data-Driven Transparency: The Case for a Food System Digital Twin

The food system is complex and diverse. This intricacy may deter and hinder transformative action. Augmenting and elaborating understanding of the system and how it operates under different conditions is an essential starting point for planning and implementing change. Accurate and transparent data are a crucial input to increase this system understanding. One way to gain insights into to how a system operates is to construct a digital twin of the system. A digital twin is generally understood to be a virtual representation of a real-world system. This might be created using real data or simulating data from samples of real data. This approach is widely used in the manufacturing and engineering sectors to monitor and respond to system errors or to maintain system levels of quality [40]. A digital twin of the food system would provide a low-cost testbed for interventions at scale, providing oversight and understanding of the systemic operations of the system that can inform actions and interventions to maximise win–wins and minimise trade-offs. This can provide significant benefits and insights, permitting near-real-time assessments of the impacts of emergent systems events (e.g., weather, geopolitical, or supply chain events) or the modelling of policy-driven food system interventions. A range of different analytical methods can be applied to the digital twin to generate insight of hypothetical interventions or policies and support decision making. Compared to randomised controlled trials, this can provide a lower-cost and faster alternative without participant burden and long delays in recruitment. As models become more sophisticated, supported by advances in data inputs, computing power, and artificial intelligence (AI), greater accuracy and granularity will be possible to model the complex interactions, feedback loops, and outcomes of multiple simultaneous events, interventions, and stressors acting across the diverse stakeholders and operations of the food system. Next-generation digital models of the food system, driven by real-world data inputs capturing food production, yields, transportation, processing, consumption, and waste, can be used to better understand the food system, build resilience, respond to system shocks, and plan for transformative change [40].

There are technical barriers to overcome. The availability of accurate data on the food system lags behind other domains. For example, the EU is already funding the development of digital twins of Earth systems to tackle climate change and protect natural environments [41]. Relevant data may not yet be routinely collected and will vary with location, food type, and stage of processing. Food companies may also be reluctant to permit access to crucial proprietary data. The UK Food Data Transparency Partnership previously announced in the GFS is a positive first step to permit greater insight and oversight into the food system as it will promote and legislate data sharing within the food system. The proposed expansion of this initiative to cover nutrition and health metrics would be a critical step to permit the modelling of food system outcomes and value not currently captured by conventional finance or consumption driven accounting methods. 

The complexity of the task should not deter action towards building the infrastructure and processes needed for a food system digital twin. There are sector-specific examples of the employment of digital twins to optimise specific food system operations, including yield forecasting [42], land use [43], and animal husbandry [44]. However, these applications are often, and necessarily, context/domain-specific as the emergent technologies and applications are developed and evaluated. Greater investment is needed to support the application of advancements in digital and AI technologies in food system sectors. Multidisciplinary collaborations between computer science and food system stakeholders (e.g., food producers, supply chain actors, food retailers, and food and nutrition experts) are essential [45]. As a first step, the scale of a digital twin should be considered. Our food system is global, yet data availability, governance structures, and technology differ dramatically between countries. Given the complexity of the food system, limiting the spatial scale to a single region or country is a pragmatic starting point for a food system digital twin, with the ambition to scale up. Government investment in this sector is not without precedent. The previous Conservative government funded a digital revolution in the built environment sector as part of a Digital Built Britain Strategy. The Centre for Digital Built Britain pioneered digital data analytics and smart technologies to promote transformation of the construction sector. Similar investment is needed in food system sectors if the transformative potential of digital technologies is to be realised [46].

**Policy Recommendation:** Investment in cross-sectorial research and innovation to advance knowledge, skills, and methodologies needed to refine and integrate digital food system approaches.

### 5.2. A Citizen Forum: Adding the Citizens’ Voice to Food System Ambitions

Engagement with food system stakeholders should be at the agenda setting stage and ongoing in the development and implementation of a food strategy. The GFS states the Government intends to “champion a collaborative approach by working in partnership with industry and civil society” (GFS; Introduction; point 13; [2]). This is commended and considered an essential approach to the success of the food strategy. The transformative change of a food system requires greater engagement and insight from those for which it functions to feed. The Department for Energy Security and Net Zero (formerly the Department for Business, Energy and Industrial Strategy [BEIS]) public attitudes tracker for climate change provides critical insight into public attitudes to environmental issues that can inform and influence climate change policy. A similar approach can provide valuable insight in other areas of the food system to inform policy. 

Such engagement can develop understanding of public attitudes to sustainability and food to help identify effective approaches to framing the need for action and subsequent policies, but transformative change needs active engagement with citizens to not only inform but to drive change in the food system. Consultation is not engagement, and strategy should involve different stakeholder groups and targeted audiences from the start to capture shared goals and values. Indeed, this is taking place independent of government action. Initiatives such as the National Conversation About Food (https://www.nationalfoodconversation.uk/, accessed on 15 January 2024) and People’s Food Policy (https://www.peoplesfoodpolicy.org/, accessed on 10 August 2024) are putting the voice of the citizen at the heart of understanding what we want from the food system. Such initiatives acknowledge it takes more than evidence alone to affect societal change; change requires support and participation from the population. Including citizens in the transformation of the food system can help address some of the key tensions previously outlined. Concerted effort and ‘buy in’ from the population on a wide scale is needed if we are to ‘break the junk food cycle’ as proposed by the NFSIR. Involving citizens in the progression towards a healthier and more equitable food system can promote a shared responsibility between government, industry, and citizens to tackle the problems faced. Engaging with citizens to understand attitudes to sustainability and diet, and developing strategies with those served by the food system, is critical for both the success and longevity of any transformative change.

The direct engagement and inclusion of citizen voices will enrich and guide transformative change, providing valuable insight and ideas for government and the food industry. Citizens ‘voting’ with their food choices is not a meaningful opportunity to contribute to the shaping the food system. Nor is it sufficient in food environments often lacking choice and inequitable access to high-quality nutrition. Including citizens’ voices in transforming the food system will ensure a wide range of perspectives—socioeconomic, cultural, geographic—are heard and specific needs related to food affordability, accessibility, and cultural preferences are acknowledged; this will give voice to marginalised or under-represented groups and contribute to efforts to promote food justice and equity. The Good Food Nation Act [47] in Scotland serves as a comparative example of national food strategy framework based on close integration of civil society and government. A key driver of this Act was the civil society group Scottish Food Coalition (https://www.foodcoalition.scot/, accessed on 20 August 2024) that campaigned for and advised government on whole food systems approaches with food justice at their heart. Wales has engagement with civil society legislatively enshrined in the Well-being of Future Generations Act [48], requiring government bodies to work collaboratively with stakeholders—including community groups and citizens—to plan long-term policies.

Engagement could also be a channel for innovation and solutions to food system challenges. Citizens may have greater insight of the food system and food environments as experienced by the average member of society compared to those in government. Further, citizen engagement can promote accountability and transparency; ensuring decisions made about the food system are linked to the interests and preferences of citizens. This can foster less suspicion and bolder action. 

**Policy Recommendation:** Formalise the engagement and contribution of citizens’ voices in establishing food systems policies and evaluation. 

### 5.3. Valuing the Food System: The Need for Greater Governance and Coherence

The challenges facing the food system demand greater prioritisation and cohesion within government departments with a responsibility for food to grasp the opportunity to enact and drive forward transformative change via a national food strategy. The lack of ambition, urgency, and coherence of the GFS for shaping the future food system is perhaps a reflection of the lack of coherence and prioritisation of food within different government departments. The NFSIR highlighted the complexity and dispersion of responsibility for food policy in England across government departments and agencies [33]. The current number of departments across which policy responsibility for the interconnected components of the food system is spread is staggering. There is no single source of food-related policy information accessible from within or outside government, no national food policy, or ministry with sole oversight of food.

The complexity of the workings and challenges facing the food system means it is inevitable there will be instances of policy responsibility spanning many areas of government. For example, the sustainability of our food system comprises consideration of a diverse, interconnected web of global food system factors, stakeholders, inputs, and outcomes. This complexity will be reflected in the array of government agencies and actors on which responsibility will fall. However, the current lack of oversight and fragmentation of responsibility must increase the possibility of policy incoherence, incompatibility, and missed opportunities for different policies to complement each other towards a wider policy goal. There is a crucial need for analysis of the coherence between the widely dispersed and often siloed food policies. Distinguishing if the likely outcomes of these interactions are complementary or antagonistic is critical to inform greater coherence on future food policy. 

Greater structure, cohesion, and prioritisation of food and food policy are essential to bring government departments together to work cohesively on coordinated long-term transformative change across the food system. The previous Government acknowledged that policy levers that influence the food system are dispersed across government and had plans to “join-up within government to collectively drive progress” (GFS; Introduction, point 13; [2]). There was no indication that this would involve any change to the way policy is currently formed or driven within or across government agencies. More ambition and coherent modes of driving change are needed, and we encourage the incoming Labour Government to address the lack of cohesion within government to coordinate and drive ambitious system change. Action should be taken to identify policy coherence failures in the food system to mitigate misalignments and bottlenecks caused by government departments silo working on cross-cutting issues. A unified, system approach, driven and galvanised by a clear and ambitious national food strategy, is essential. This requires greater prioritisation of food and cohesion of those responsible for food and the food system within government. 

Food system transformation requires long-term vision, focus, and political momentum; these are difficult to maintain across electoral cycles, changes in political priorities, and the fragmentation of policy into silos. The NFSIR proposed a governance approach to overcome this disconnect between government cycles and long-termism: a Good Food Bill that would set long-term statutory targets to improve dietary health and define the governance structures to meet targets [5]. The Food Standards Agency, as an independently governed non-ministerial government department, was recommended to oversee and monitor the progress of long-term strategies promoting food system approaches to healthy and sustainable food. The Government would be required to publish a Good Food Action Plan every 5 years, which would be subject to independent review (by the FSA to Parliament) to incentivise Governments to keep long-term commitments on national progress towards a healthy, sustainable food system. A comparable approach has been adopted in Scotland, which is on course to be the first UK nation to have a systems approach-centric food plan. The Good Nation Food Bill outlines statutory duties for the Scottish Government to establish cross-cutting National Food Plans employing strategic, joined-up policy-making. Local authorities and Health Boards will be required to produce systems-level Food Action Plans to meet food system targets [47]. These targets will be overseen by an independent Food Commission to foster continued policy momentum.

Another approach to increase food policy coherence is to adopt a cross-cutting policy or regulatory benchmark that is adhered to or incorporated into relevant policy and strategy planning. To promote a more consistent approach across government policy, the NFSIR recommended the adoption of a healthy and sustainable reference diet to which all public bodies would refer to inform food-related policy and public procurement [5]. Such a reference diet is employed in the USA to consistently inform policy across all state-funded schemes (e.g., the National School Lunch Programme). Finland has long promoted policy coherence across siloed government ministries through ‘Health in All Policies’ (HiAP). Target health objectives are included in policy planning and decision making across government ministries (e.g., transport, trade, agriculture) to promote policy coherence [49].

Enacting a coherent, transformative food strategy is no easy task. Numerous food strategies introduced by other nations have been criticised for focusing on discrete elements of the food system rather than the whole, and promoting incremental rather than transformative change [50,51,52]. Adopting the concepts of ‘directionality’ and ‘reflexivity’ can help promote coherent, transformative food system change [50,53]. Directionality refers to an agreed long-term vision for a sustainable food system. Reflexivity refers to the governance tools and methods to engage food system stakeholders to monitor, evaluate, learn and respond [50]. A coherent national food strategy based on the collective, balanced interests of food system stakeholders—including sustainable environmental boundaries—is a critical starting point for transformative change. We encourage the incoming government to reconsider the recommendations of NFSIR and deliver a coherent, truly transformative national food strategy to serve as a long-term vision for a sustainable future. As per our previous recommendations, greater engagement with stakeholders—particularly citizens—and investing in digital solutions to extend oversight of, and feedback from, the food system will increase the reflexivity needed to inform, monitor and respond to the challenge of enacting transformative change. 

**Policy Recommendation**: Reassessment of the NFSIR to inform the creation of a national food strategy with greater coherence and transformative ambition.

## 6. Conclusions

The UK food system is a national asset delivering cultural, economic, social, health, and nutritional value. However, alignment of the needs and priorities of food system stakeholders with deeply vested, and often disparate, interests in accessing this value requires coherent and ambitious food system governance to ensure the food system delivers for all. The GFS as envisaged by the outgoing Conservative Government lacked this coherence and ambition in its aspirations. To reduce fragmentation of interest and build a transformative future demands a systems approach with coordinated action at multiple levels of the food system that acknowledges and optimises the balance between stakeholders’ interests and the integrity of our ecosystems. We propose the incumbent Labour UK Government puts forward a truly transformative, bold food strategy that can serve as a catalyst for wider action towards this lofty, yet imperative, objective. The impacts of the global pandemic and geo-political uncertainty on food security have highlighted the importance of systems thinking to co-produce safe, resilient, and healthy food. Interest and willingness to adopt change may never by greater than in the wake of the global, seismic events of recent years. It is imperative to grasp opportunity from chaos and harness appetite for change. 

## Data Availability

No new data were created or analysed in this study. Data sharing is not applicable to this article.
